# Dynamic Instability of Functionally Graded Graphene Platelet-Reinforced Porous Beams on an Elastic Foundation in a Thermal Environment

**DOI:** 10.3390/nano12224098

**Published:** 2022-11-21

**Authors:** Jing Zhang, Ying Lv, Lianhe Li

**Affiliations:** 1 College of Mathematics Science, Inner Mongolia Normal University, Hohhot 010022, China; 2 Inner Mongolia Center of Applied Mathematics, Hohhot 010022, China

**Keywords:** graphene nanocomposite, functionally graded porous beams, free vibration, thermal buckling, dynamic instability, elastic foundation

## Abstract

Under thermal environment and axial forces, the dynamic instability of functionally graded graphene platelet (GPLs)-reinforced porous beams on an elastic foundation is investigated. Three modes of porosity distributions and GPL patterns are considered. The governing equations are given by the Hamilton principle. On the basis of the differential quadrature method (DQM), the governing equations are changed into Mathieu–Hill equations, and the main unstable regions of the porous composite beams are studied by the Bolotin method. Thermal buckling and thermo-mechanical vibration problems are also studied. The effects of porosity coefficients and GPL weight fraction, dispersion pattern, initial thermal loading, slenderness ratio, geometry and size, boundary conditions, and foundation stiffness are discussed. The conclusions show that an elastic foundation has an obvious enhancement effect on thermal buckling, free vibration, and dynamic instability, which improves the stiffness of the beam.

## 1. Introduction

Many natural porous materials have been widely used for thousands of years. Compared with continuous medium materials, porous materials have excellent impact resistance, electrical conductivity, energy absorption, and thermal management properties [1,2,3,4,5]. Porous materials are often used in biological tissue, sound insulation materials, and new photoelectric elements [6]. As a kind of porous material, metal foam has high strength and stiffness [7]. At present, many scholars have probed into the mechanical behavior of porous materials and the influence of various factors on the materials.

Nguyen et al. [8] investigated the bucking, bending, and vibration of functionally graded porous (FGP) beams by the Ritz method. Akbaş [9,10,11] examined bucking and vibration by the finite element method. By the differential transform method (DTM), Ebrahimi and Mokhtari [12] presented the vibration of rotating FGP beams. Rjoub and Hamad [13] reported on the vibration of FGP beams by the Transfer Matrix Method. Wattanasakulpong and Chaikittiratana [14] found that a uniform distribution of porosities has an obvious effect on natural frequencies. Chen et al. [15,16,17] studied the buckling and bending of shear-deformable FGP beams by the Ritz method. Hoa et al. [18] studied nonlinear buckling and post-buckling of cylindrical shells by the three-terms solution and Galerkin’s method. Under various boundary conditions, Chan et al. [19] discussed nonlinear buckling and post-buckling of imperfect FG porous sandwich cylindrical panels by Galerkin’s method.

In order to meet the high efficiency of civil engineering structures and the high precision development of aerospace engineering devices, beams need to be thinner and thinner, and at the same time, it is necessary to improve the material strength of its structure so as to increase the effective space and load. Nanomaterials have good mechanical, thermal, optical, and electrical properties [20], so carbon nanomaterials are often regarded as nanofillers to heighten matrix materials’ properties. These include graphene platelets [21], carbon nanotubes [22], and fullerenes. In 2004, British scientists were the first to peel graphene sheets from graphite with an extremely high tensile, Young modulus, and surface area [23]. On account of the porous structure of metal foam, the stiffness and strength are weakened compared with that of dense metal. By filling the carbon nanomaterials into the matrix materials, the properties of porous materials are able to be efficiently improved. At present, there have been many studies on graphene-reinforced porous materials.

Many papers have been published on graphene-reinforced porous composite beams, plates, and shells. Kitipornchai et al. [24,25] investigated the static and dynamic mechanical behavior of graphene-reinforced FGP beams. Yas et al. [26,27] presented the buckling and vibration of graphene-reinforced FGP beams in thermal environments. Yang et al. [28] studied the buckling and vibration of graphene-reinforced FGP plates. Teng and Wang [29] explained the nonlinear forced vibration of simply supported graphene-reinforced FGP plates. Dong et al. [30] researched the buckling of spinning graphene-reinforced FGP shells. By Galerkin’s method, Zhou et al. [31] revealed the nonlinear buckling of graphene-reinforced FGP cylindrical shells. Under impulsive loading, Yang et al. [32] studied nonlinear forced vibration and the dynamic buckling of graphene-reinforced FGP arches.

Graphene-reinforced composite porous beams and plates are easily affected by the thermal environment, resulting in a decrease in their structural stiffness. Therefore, it is of great significance to study their thermal buckling, free vibration, and dynamic instability for engineering practices. To the best of the authors’ knowledge, no relevant literature has studied the dynamic instability of graphene-enhanced porous materials based on elastic foundations, thermal environments, and axial forces. The present paper mainly investigates the dynamic instability, thermal buckling, and free vibration of functionally graded graphene platelet-reinforced porous beams on an elastic foundation under a thermal environment and axial forces. Three modes of GPL patterns and porosity distributions are considered. Based on the theory of the Timoshenko beam, the governing equation is obtained by the Hamilton principle. On the basis of the differential quadrature method (DQM), the governing equations are changed into Mathieu–Hill equations and the main unstable regions of porous composite beams are studied by the Bolotin method. Moreover, we also use the two-step perturbation method (TSPM) to calculate the thermal buckling and free vibration. The effects of porosity coefficients and GPL’s weight fraction, initial thermal loading, slenderness ratio, geometry and size, boundary conditions, foundation stiffness, and dispersion pattern are discussed.

## 2. Model Construction

Under axial force $N_{x_{0}}$ and uniform temperature change $\Delta T=T-T_{0}$, we consider a FGP multilayer beam that rests on a two-parameter elastic foundation in an initial stress-free state at the reference temperature $T_{0}$.

**Figure 1:**
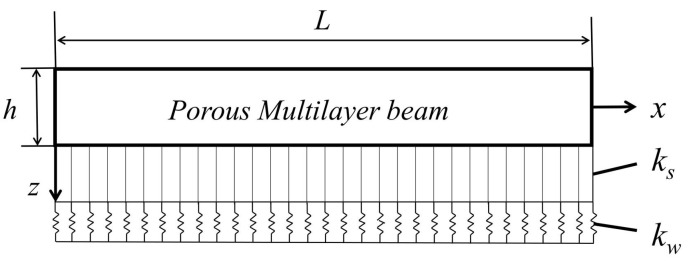
FG porous multilayer beam resting on an elastic foundation.

As seen in Figure 1, *L*, *b* and *h*, respectively, represent the length, width, and thickness of the beam, and $k_{w}$ and $k_{s}$ are the Winkler stiffness and shearing layer stiffness. Among others, the thickness *h* is divided into n layers, each of which is ▵h=h/n.

**Figure 2:**
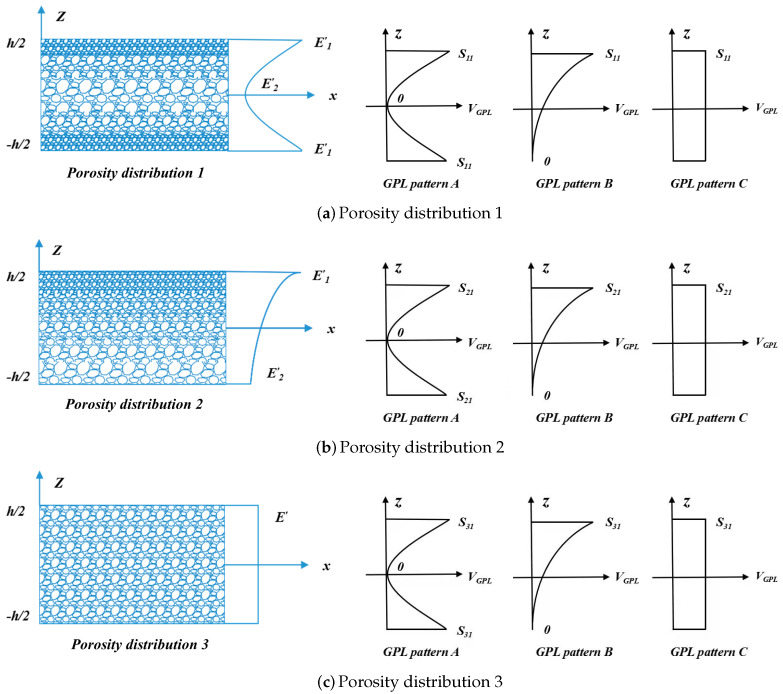
GPL patterns and porosity distributions.

Figure 2 considers three porosity distributions and GPL patterns. Because of disparate dispersion patterns, GPL patterns can be divided as A, B, and C, and the GPLs volume content $V_{GPL}$ is smoothly on the z-axis. According to different porosity distributions, $V_{GPL}$’s peak values can be denoted as $s_{ij}\left(i,j=1,2,3\right)$. Assuming three GPL patterns have the same total amount of GPLs will result in $s_{1i}\neq s_{2i}\neq s_{3i}$.

$E_{1}^{\prime }$ and $E_{2}^{\prime }$ are denoted as the maximum and minimum elastic moduli of the non-uniform porous beams without GPLs, respectively. In addition, $E^{\prime }$ represents the elastic moduli of the uniform porosity distribution beams.

The relationships of elastic moduli E(z), mass density ρ(z), and thermal expansion coefficient α(z) of FGP beams for three porosity distributions are given by the following formulas [25,33]

(1) $$\left\{\begin{array}{c} E\left(z\right)=E_{1}\left[1-e_{0}\lambda \left(z\right)\right],\\ \rho \left(z\right)=\rho_{1}\left[1-e_{m}\lambda \left(z\right)\right],\\ G(z)=E(z)/2[1+\nu (z)],\\ \alpha \left(z\right)=\alpha_{1} \end{array}\right.$$

where

(2) $$\lambda \left(z\right)=\left\{\begin{array}{cc} cos(\pi \beta ) & Porosity\;distribution\;1\\ cos\left(\pi \beta /2+\pi /4\right) & Porosity\;distribution\;2\\ \lambda^{*} & Porosity\;distribution\;3 \end{array}\right.$$

in which β=z/h. Further, $E_{1}$, $\rho_{1}$, and $\alpha_{1}$ are the maximum values of E(z), ρ(z), and α(z), respectively. The porosity coefficient $e_{0}$ is referred to as

(3) $$e_{0}=1-\frac{E_{2}^{\prime }}{E_{1}^{\prime }},$$

Based on the Gaussian Random Field (GRF) scheme, the mechanical property of closed-cell cellular solids is denoted by [34]

(4) $$\frac{E(z)}{E_{1}}={\left(\frac{\rho \left(z\right)/\rho_{1}+0.121}{1.121}\right)}^{2.3},\left(0.15<\frac{\rho (z)}{\rho_{1}}<1\right)$$

Using Equation (4), the coefficient of mass density $e_{m}$ is given by the following formula

(5) $$e_{m}=\frac{1.121(1-\sqrt[{2.3}]{1-e_{0}\lambda \left(z\right)})}{\lambda (z)},$$

Similarly, using the closed-cell GRF scheme, Poisson’s ratio ν(z) is defined as [35]

(6) $$\nu \left(z\right)=0.221p^{\prime }+\nu_{1}\left({0.342p\prime }^{2}-1.21p^{\prime }+1\right),$$

where $\nu_{1}$ is Poisson’s ratio of pure non-porous matrix materials and

(7) $$p^{\prime }=1-\frac{\rho (z)}{\rho_{1}}=1.121\left(1-\sqrt[{2.3}]{1-e_{0}\lambda \left(z\right)}\right),$$

Due to the total masses being the same for the three porosity distributions, $\lambda^{*}$ in Equation (2) can be defined as

(8) $$\lambda^{*}=\frac{1}{e_{0}}-\frac{1}{e_{0}}{\left(\frac{M/\rho_{1}h+0.121}{1.121}\right)}^{2.3},$$

in which *M* represents all porosity distributions, as shown in the following equation

(9) $$M=\int_{-h/2}^{h/2}\rho_{1}\left(1-p^{\prime }\right)dz,$$

According to the distribution patterns, the volume fraction of GPLs $V_{GPL}$ is denoted by

(10) $$V_{GPL}=\left\{\begin{array}{cc} s_{i1}\left[1-cos\left(\pi \beta \left(z\right)\right)\right] & GPL\;pattern\;A\\ s_{i2}\left[1-cos\left(\pi \beta \left(z\right)/2+\pi /4\right)\right] & GPL\;pattern\;B\\ s_{i3} & GPL\;pattern\;C \end{array}\right.$$

in which i=1,2,3.

The relationship between the weight fraction of GPLs $\Lambda_{GPL}$ and the volume fraction of GPLs $V_{GPL}$ is given by

(11) $$\frac{\Lambda_{GPL}}{\Lambda_{GPL}+\frac{\rho_{GPL}}{\rho_{M}}-\frac{\rho_{GPL}}{\rho_{M}}\Lambda_{GPL}}\int_{-h/2}^{h/2}\left[1-e_{m}\lambda \left(z\right)\right]dz=\int_{-h/2}^{h/2}V_{GPL}\left[1-e_{m}\lambda \left(z\right)\right]dz$$

Based on Halpin–Tsai micromechanics model [36,37,38,39], the elastic moduli $E_{1}$ of the nanocomposites is defined as

(12) $$E_{1}=\frac{3}{8}\left(\frac{1+\xi_{L}^{GPL}\eta_{L}^{GPL}V_{GPL}}{1-\eta_{L}^{GPL}V_{GPL}}\right)E_{M}+\frac{5}{8}\left(\frac{1+\xi_{W}^{GPL}\eta_{W}^{GPL}V_{GPL}}{1-\eta_{W}^{GPL}V_{GPL}}\right)E_{M}$$

where

(13) $$\begin{array}{c} \xi_{L}^{GPL}=2\frac{a_{GPL}}{t_{GPL}},\xi_{W}^{GPL}=2\frac{b_{GPL}}{t_{GPL}}\\ \eta_{L}^{GPL}=\frac{(E_{GPL}/E_{m})-1}{\left(E_{GPL}/E_{M}\right)+\xi_{L}^{GPL}},\\ \eta_{W}^{GPL}=\frac{(E_{GPL}/E_{m})-1}{\left(E_{GPL}/E_{M}\right)+\xi_{W}^{GPL}}. \end{array}$$

where $a_{GPL}$, $b_{GPL}$, and $t_{GPL}$ are the average length, width, and thickness of GPLs. $E_{M}$ and $E_{GPL}$ represent the elastic moduli of the metal and GPLs.

By the following mixture rule, the mass density $\rho_{1}$, Poisson’s ratio $\upsilon_{1}$, and thermal expansion coefficient $\alpha_{1}$ of the metal matrix reinforced by GPLs can be obtained as

(14) $$\begin{array}{cc} \rho_{1} & =\rho_{GPL}V_{GPL}+\rho_{M}V_{M},\\ \upsilon_{1} & =\upsilon_{GPL}V_{GPL}+\upsilon_{M}V_{M},\\ \alpha_{1} & =\alpha_{GPL}V_{GPL}+\alpha_{M}V_{M}. \end{array}$$

in which $\rho_{M}$, $\upsilon_{M}$, $\alpha_{M}$, and $V_{M}=1-V_{GPL}$ are the mass density, Poisson’s ratio, thermal expansion coefficient, and volume fraction of the metals. Furthermore, $\rho_{GPL}$, $\upsilon_{GPL}$, $\alpha_{GPL}$, and $V_{GPL}$ are the corresponding properties of GPLs.

## 3. Formulations

### 3.1. Equations of Governing

Based on the Timoshenko beam theory, the displacement components are expressed as

(15) $$\left\{\begin{array}{c} \bar{U}\left(x,z,t\right)=U\left(x,t\right)+z\;\Psi \left(x,t\right),\\ \bar{W}\left(x,z,t\right)=W\left(x,t\right). \end{array}\right.$$

in which *U* and *W* are the displacements of the *x* and *z*-axes, Ψ is expressed as the normal transverse rotation of the *y*-axis, and *t* represents time. On the basis of the linear stress-displacement relationships

(16) $$\left\{\begin{array}{c} \varepsilon_{xx}=\frac{\partial U}{\partial x}+z\frac{\partial \Psi }{\partial x},\\ \gamma_{xz}=\frac{\partial W}{\partial x}+\Psi . \end{array}\right.$$

The linear stress-strain constitutive relationships are as follows

(17) $$\left\{\begin{array}{c} \sigma_{xx}=Q_{11}\left(z\right)\left[\varepsilon_{xx}-\alpha \left(z\right)\Delta T\right],\\ \sigma_{xz}=Q_{55}\left(z\right)\gamma_{xz}. \end{array}\right.$$

where elastic elements $Q_{11}\left(z\right)$ and $Q_{55}\left(z\right)$ are denoted by

(18) $$\left\{\begin{array}{c} Q_{11}\left(z\right)=\frac{E(z)}{1-\upsilon^{2}\left(z\right)},\\ Q_{55}\left(z\right)=G\left(z\right). \end{array}\right.$$

Using Hamilton’s principle, the considered problems can be expressed as

(19) $$\int_{0}^{t}\delta \left(T+V-\Pi \right)dt=0$$

in which

(20) $$\begin{array}{cc} \delta T & =\int_{0}^{L}\int_{-h/2}^{h/2}\rho \left(z\right)\left[{\left(\frac{\partial \bar{U}}{\partial t}\right)}^{2}+{\left(\frac{\partial \bar{W}}{\partial t}\right)}^{2}\right]dzdx,\\ \delta V & =\int_{0}^{L}\left[\left(N_{x0}+N_{x}^{T}\right){\left(\frac{\partial W}{\partial x}\right)}^{2}\right]dx,\\ \delta \Pi & =\int_{0}^{L}\int_{-h/2}^{h/2}\left(\sigma_{xx}\varepsilon_{xx}+\sigma_{xz}\varepsilon_{xz}\right)dzdx+\int_{0}^{L}\left[k_{w}W^{2}+k_{s}{\left(\frac{\partial W}{\partial x}\right)}^{2}\right]dx. \end{array}$$

δ stands for the variational symbol, *T* is the kinetic energy of the beam, *V* is made up of the axial force $N_{x0}$ and thermally axial force $N_{x}^{T}$ due to uniform temperature change ΔT, and II consists of the strain energy of the beam and the elastic potential energy of the foundation.

The governing equations are given by applying Equation (20) to Hamilton’s principle in Equation (19), integrating through the beam thickness

(21) $$\begin{array}{cc} 0= & \int_{0}^{t}\int_{0}^{L}\left[\left(\frac{\partial N_{x}}{\partial x}-I_{1}\frac{\partial^{2}U}{\partial t^{2}}-I_{2}\frac{\partial^{2}\Psi }{\partial t^{2}}\right)\delta U\right]dxdt+\int_{0}^{t}\int_{0}^{L}\left[\left(\frac{\partial Q_{x}}{\partial x}-k_{w}W+k_{s}\frac{\partial^{2}W}{\partial x^{2}}-\left(N_{x0}+N_{x}^{T}\right)\frac{\partial^{2}W}{\partial x^{2}}-I_{1}\frac{\partial^{2}W}{\partial t^{2}}\right)\delta W\right]dxdt\\ \; & +\int_{0}^{t}\int_{0}^{L}\left[\left(\frac{\partial M_{x}}{\partial x}-Q_{x}-I_{2}\frac{\partial^{2}U}{\partial t^{2}}-I_{3}\frac{\partial^{2}\Psi }{\partial t^{2}}\right)\delta \Psi \right]dxdt-\int_{0}^{t}\left[\left(N_{x}\delta U\right){|}_{0}^{L}+\left(M_{x}\delta \Psi \right){|}_{0}^{L}-\left(Q_{x}-N_{x0}\frac{\partial W}{\partial x}-N_{x}^{T}\frac{\partial W}{\partial x}+k_{s}\frac{\partial W}{\partial x}\right)\delta W{|}_{0}^{L}\right]dt \end{array}$$

Letting coefficients δU, δW, and δΨ from Equation (21) go to zero separately,

(22) $$\begin{array}{cc} \; & \frac{\partial N_{x}}{\partial x}=I_{1}\frac{\partial^{2}U}{\partial t^{2}}+I_{2}\frac{\partial^{2}\Psi }{\partial t^{2}},\\ \; & \frac{\partial Q_{x}}{\partial x}-k_{w}W+k_{s}\frac{\partial^{2}W}{\partial x^{2}}-\left(N_{x0}+N_{x}^{T}\right)\frac{\partial^{2}W}{\partial x^{2}}=I_{1}\frac{\partial^{2}W}{\partial t^{2}},\\ \; & \frac{\partial M_{x}}{\partial x}-Q_{x}=I_{2}\frac{\partial^{2}U}{\partial t^{2}}+I_{3}\frac{\partial^{2}\Psi }{\partial t^{2}}. \end{array}$$

the force and moment are referred to

(23) $$\left\{\begin{array}{c} N_{x}\\ M_{x}\\ Q_{x} \end{array}\right\}=\int_{-h/2}^{h/2}\left\{\begin{array}{c} \sigma_{xx}\\ z\sigma_{xx}\\ \sigma_{xz} \end{array}\right\}dz$$

Applying Equations (17) and (18) to Equation (23),

(24) $$\begin{array}{cc} N_{x} & =A_{11}\frac{\partial U}{\partial x}+B_{11}\frac{\partial \Psi }{\partial x}-N_{x}^{T},\\ M_{x} & =B_{11}\frac{\partial U}{\partial x}+D_{11}\frac{\partial \Psi }{\partial x}-M_{x}^{T},\\ Q_{x} & =\kappa A_{55}\left(\frac{\partial W}{\partial x}+\Psi \right). \end{array}$$

where κ=5/6 denotes the shear correction factor. The stiffness components, inertia terms, and thermally induced force and moment are defined as

(25) $$\begin{array}{cc} \; & \left(A_{11},B_{11},D_{11}\right)=\int_{-h/2}^{h/2}Q_{11}\left(z\right)\left(1,z,z^{2}\right)dz=\frac{h}{n}\sum_{i=1}^{n}\frac{E_{1}\left(h\beta_{i}\right)\left[1-e_{0}\lambda \left(h\beta_{i}\right)\right]}{1-\nu^{2}\left(h\beta_{i}\right)}\left(1,h\beta_{i},{\left(h\beta_{i}\right)}^{2}\right),\\ \; & A_{55}=\int_{-h/2}^{h/2}Q_{55}\left(z\right)dz=\frac{h}{n}\sum_{i=1}^{n}\kappa \frac{E_{1}\left(h\beta_{i}\right)\left[1-e_{0}\lambda \left(h\beta_{i}\right)\right]}{2[1+\nu \left(h\beta_{i}\right)]},\\ \; & \left(I_{1},I_{2},I_{3}\right)=\int_{-h/2}^{h/2}\rho \left(z\right)\left(1,z,z^{2}\right)dz=\frac{h}{n}\sum_{i=1}^{n}\rho_{1}\left(h\beta_{i}\right)\left[1-e_{m}\lambda \left(h\beta_{i}\right)\right]\left(1,h\beta_{i},{\left(h\beta_{i}\right)}^{2}\right),\\ \; & \left(N_{x}^{T},M_{x}^{T}\right)=\int_{-h/2}^{h/2}Q_{11}\left(z\right)\alpha \Delta T\left(1,z\right)dz=\frac{h}{n}\sum_{i=1}^{n}\frac{E_{1}\left(h\beta_{i}\right)\left[1-e_{0}\lambda \left(h\beta_{i}\right)\right]}{1-\nu^{2}\left(h\beta_{i}\right)}\alpha \left(h\beta_{i}\right)\Delta T\left(1,h\beta_{i}\right). \end{array}$$

in which $\beta_{i}=\frac{1}{2}+\frac{1}{2n}-\frac{i}{n}\;\left(i=1,2,3,\ldots ,n\right)$.

The governing equations and related boundary conditions are expressed through Equations (22)–(25)

(26) $$\begin{array}{cc} \; & A_{11}\frac{\partial^{2}U}{\partial x^{2}}+B_{11}\frac{\partial^{2}\Psi }{\partial x^{2}}=I_{1}\frac{\partial^{2}U}{\partial t^{2}}+I_{2}\frac{\partial^{2}\Psi }{\partial t^{2}},\\ \; & \kappa A_{55}\left(\frac{\partial^{2}W}{\partial x^{2}}+\frac{\partial \Psi }{\partial x}\right)-k_{w}W+k_{s}\frac{\partial^{2}W}{\partial x^{2}}-\left(N_{x0}+N_{x}^{T}\right)\frac{\partial^{2}W}{\partial x^{2}}=I_{1}\frac{\partial^{2}W}{\partial t^{2}},\\ \; & B_{11}\frac{\partial^{2}U}{\partial x^{2}}+D_{11}\frac{\partial^{2}\Psi }{\partial x^{2}}-\kappa A_{55}\left(\frac{\partial W}{\partial x}+\Psi \right)=I_{2}\frac{\partial^{2}U}{\partial t^{2}}+I_{3}\frac{\partial^{2}\Psi }{\partial t^{2}}. \end{array}$$

(27) $$\begin{array}{cc} \; & Clamped(C):\;U=W=\Psi =0,\\ \; & Hinged\left(H\right):\;U=W=M_{x}=0. \end{array}$$

By introducing dimensionless quantities

(28) $$\begin{array}{cc} \; & \xi =\frac{x}{L},\;\left(u,w\right)=\frac{\left(U,W\right)}{h},\;\eta =\frac{L}{h},\;\psi =\Psi ,\;\left(P,P^{T},M^{T}\right)=\left(N_{x0},N_{x}^{T},M_{x}^{T}/h\right)/A_{110},\\ \; & \left({\bar{I}}_{1},{\bar{I}}_{2},{\bar{I}}_{3}\right)=\left(\frac{I_{1}}{I_{10}},\frac{I_{2}}{I_{10}h},\frac{I_{3}}{I_{10}h^{2}}\right),\left(a_{11},a_{55},b_{11},d_{11}\right)=\left(\frac{A_{11}}{A_{110}},\frac{A_{55}}{A_{110}},\frac{B_{11}}{A_{110}h},\frac{D_{11}}{A_{110}h^{2}}\right),\\ \; & \;K_{w}=\frac{k_{w}L^{2}}{A_{110}},\;K_{s}=\frac{k_{s}}{A_{110}},\omega =\Omega L\sqrt{\frac{I_{10}}{A_{110}}},\;\tau =\frac{t}{L}\sqrt{\frac{A_{110}}{I_{10}}}. \end{array}$$

in which $A_{110}$ and $I_{10}$ are the value of $A_{11}$ and $I_{1}$ of the pure metal beams without any pores and nanofillers.

Then Equations (26) and (27) are rewritten into the following dimensionless form

(29) $$\begin{array}{cc} \; & a_{11}\frac{\partial^{2}u}{\partial \xi^{2}}+b_{11}\frac{\partial^{2}\psi }{\partial \xi^{2}}={\bar{I}}_{1}\frac{\partial^{2}u}{\partial \tau^{2}}+{\bar{I}}_{2}\frac{\partial^{2}\psi }{\partial \tau^{2}},\\ \; & \kappa a_{55}\left(\frac{\partial^{2}w}{\partial \xi^{2}}+\eta \frac{\partial \psi }{\partial \xi }\right)-K_{w}w+K_{s}\frac{\partial^{2}w}{\partial \xi^{2}}-\left(P+P^{T}\right)\frac{\partial^{2}w}{\partial \xi^{2}}={\bar{I}}_{1}\frac{\partial^{2}\bar{w}}{\partial \tau^{2}},\\ \; & b_{11}\frac{\partial^{2}u}{\partial \xi^{2}}+d_{11}\frac{\partial^{2}\psi }{\partial \xi^{2}}-\kappa \eta a_{55}\left(\frac{\partial w}{\partial \xi }+\eta \psi \right)={\bar{I}}_{2}\frac{\partial^{2}u}{\partial \tau^{2}}+{\bar{I}}_{3}\frac{\partial^{2}\psi }{\partial \tau^{2}}. \end{array}$$

(30) $$\begin{array}{cc} \; & Clamped(C):u=w=\psi =0,\\ \; & Hinged\left(H\right):u=w=b_{11}\frac{\partial u}{\partial \xi }+d_{11}\frac{\partial \psi }{\partial \xi }-M^{T}=0. \end{array}$$

### 3.2. Solution Method

Based on the DQM rule, the displacement components u,w, and ψ, and the rth-order partial derivative is estimated in the following form [40,41]

(31) $$\begin{array}{cc} \; & \left(u,w,\psi \right)=\sum_{m=1}^{N}l_{m}\left(\xi \right)\left(u_{m},w_{m},\psi_{m}\right),\\ \; & \frac{\partial^{j}}{\partial \xi^{j}}\left(u,w,\psi \right)=\sum_{m=1}^{N}c_{im}^{\left(j\right)}\left(u_{m},w_{m},\psi_{m}\right),\;i=1,\ldots ,N,\;j=1,\ldots ,N-1. \end{array}$$

in which $\left(u_{m},w_{m},\psi_{m}\right)$ are the values of (u,w,ψ), and $l_{m}\left(\xi \right)$ is the Lagrange interpolating polynomial; $c_{im}^{\left(j\right)}$ is the weighting coefficient of the jth-order derivative [42]. *N* is the total number of grid points along the ξ-axis. The distribution of the ξ-axis is defined by a cosine pattern

(32) $$\xi_{i}=\frac{1}{2}\left[1-cos\left(\frac{i-1}{N-1}\pi \right)\right],\;i=1,2,\ldots ,N.$$

By taking Equations (31) and (32) into Equations (29) and (30), the governing equations and boundary conditions are written as

(33) $$\begin{array}{cc} \; & a_{11}\sum_{m=1}^{N}C_{im}^{\left(2\right)}u_{m}+b_{11}\sum_{m=1}^{N}C_{im}^{\left(2\right)}\psi_{m}={\bar{I}}_{1}\ddot{u_{i}}+{\bar{I}}_{2}\ddot{\psi_{i}},\\ \; & \kappa a_{55}\left(\sum_{m=1}^{N}C_{im}^{\left(2\right)}w_{m}+\eta \sum_{m=1}^{N}C_{im}^{\left(1\right)}\psi_{m}\right)-K_{w}w_{i}+K_{s}\sum_{m=1}^{N}C_{im}^{\left(2\right)}w_{m}-\left(P+P^{T}\right)\sum_{m=1}^{N}C_{im}^{\left(2\right)}w_{m}={\bar{I}}_{1}\ddot{w_{i}},\\ \; & b_{11}\sum_{m=1}^{N}C_{im}^{\left(2\right)}u_{m}+d_{11}\sum_{m=1}^{N}C_{im}^{\left(2\right)}\psi_{m}-\kappa \eta a_{55}\left(\sum_{m=1}^{N}C_{im}^{\left(1\right)}w_{m}+\eta \psi_{i}\right)={\bar{I}}_{2}\ddot{u_{i}}+{\bar{I}}_{3}\ddot{\psi_{i}}. \end{array}$$

(34) $$\begin{array}{cc} \; & \;u_{1}=w_{1}=\psi_{1}=0,\\ \; & \;u_{N}=w_{N}=\psi_{N}=0. \end{array}$$

clamped at both ends of ξ=0,1.

(35) $$\begin{array}{cc} \; & \;u_{1}=w_{1}=b_{11}\sum_{m=1}^{N}C_{1m}^{\left(1\right)}u_{m}+d_{11}\sum_{m=1}^{N}C_{1m}^{\left(1\right)}\psi_{m}-\eta M^{T}{|}_{\xi =\xi_{1}}=0,\\ \; & \;u_{N}=w_{N}=b_{11}\sum_{m=1}^{N}C_{Nm}^{\left(1\right)}u_{m}+d_{11}\sum_{m=1}^{N}C_{Nm}^{\left(1\right)}\psi_{m}-\eta M^{T}{|}_{\xi =\xi_{N}}=0. \end{array}$$

hinged at both ends of ξ=0,1, where $C_{im}^{\left(1\right)}$ and $C_{im}^{\left(2\right)}$ represent the first- and second-order weighting coefficients.

By combining the discretized governing equation, Equation (33), and boundary conditions, Equations (34) and (35), a series of dimensionless algebraic formulas has been obtained as

(36) $$M\ddot{d}+\left(K_{L}-\Delta TK_{T}-PK_{p}\right)d=0$$

where $d={\left\{\left\{u_{1},u_{2},\ldots ,u_{N}\right\},\left\{w_{1},w_{2},\ldots ,w_{N}\right\},\left\{\psi_{1},\psi_{2},\ldots ,\psi_{N}\right\}\right\}}^{T}$ is the unknown coefficient vector, $K_{L}$ and M represent the stiffness matrix and the mass matrix, and $K_{T}$ and $K_{p}$ represent the geometric stiffness matrix.

For the beam under a time-varying axial excitation, the dimensionless axial force *P* is defined as

(37) $$P=P_{s}+P_{d}\cos\theta \tau$$

in which $P_{s}$ and $P_{d}$ represent the static and dynamic force components. By putting Equation (37) into Equation (36), we have

(38) $$M\ddot{d}+\left[K_{L}-\Delta TK_{T}-\left(P_{s}+P_{d}\cos\theta \tau \right)K_{p}\right]d=0$$

Under axial force and initial thermal loading, Equation (38) is a Mathieu–Hill-type equation, which is used to solve the problems of dynamic instability of the FGP beams. The boundary of the unstable region is obtained by Bolotin’s method [43]. According to the previous study, the solution with period 2 $T_{\theta }$ ($T_{\theta }=2\pi /\theta$) has a larger principal unstable region than the solution with period $T_{\theta }$, which is closer to the practical engineering significance. The solution to Equation (38) with period 2 $T_{\theta }$ uses the trigonometric form

(39) $$d=\sum_{k=1,3,..}^{\infty }a_{k}\sin\left(\frac{k\theta \tau }{2}\right)+b_{k}\cos\left(\frac{k\theta \tau }{2}\right)$$

where $a_{k}$ and $b_{k}$ are arbitrary constant vectors. Bolotin verified that the first-order approximation of k=1 accurately describes the boundary of the unstable region [43], so a homogeneous linear system of equations represented by $a_{1}$ and $b_{1}$ can be obtained by Bolotin’s method

(40) $$\begin{array}{cc} \; & \left[K_{L}-\Delta TK_{T}-\left(P_{s}-\frac{P_{d}}{2}\right)K_{p}-\frac{\theta^{2}}{4}M\right]a_{1}=0,\\ \; & \left[K_{L}-\Delta TK_{T}-\left(P_{s}+\frac{P_{d}}{2}\right)K_{p}-\frac{\theta^{2}}{4}M\right]b_{1}=0. \end{array}$$

For a given axial force, Equation (40) gives two critical excitation frequencies. The two curves in the figure of θ and $P_{d}$ are used to describe the principle unstable regions. When $P_{d}=0$, it represents the origin of the principle unstable region, and θ represents the doubled fundamental frequency of the beam.

As for the thermal buckling problem, we form the equation by neglecting the inertia terms and making $P_{s}=P_{d}=0$ from Equation (38). Thus the critical buckling temperature rise can be obtained by solving the minimum positive eigenvalue of Equation (38)

(41) $$\left(K_{L}-\Delta TK_{T}\right)d=0$$

Like thermal buckling, by setting $P_{d}=0$ and letting $d=d^{*}e^{i\omega t}$, the free frequency of the beam makes from the following formula

(42) $$\left(K_{L}-\Delta TK_{T}-P_{s}K_{p}-\omega^{2}M\right)d^{*}=0$$

## 4. Discussion

The effects of various factors on thermal buckling, thermo-mechanical vibration, and dynamic instability of FGP beams are discussed. Copper is often chosen as a matrix material, the material parameters of which are $E_{M}$ = 130 GPa, $\rho_{M}$ = 8960 kg/m${}^{3}$, $v_{M}$ = 0.34, and $\alpha_{M}$ = 17 $\times 10^{-6}$$K^{-1}$. The material and size parameters of GPLs as reinforced materials are $w_{GPL}$ = 1.5 μm, $l_{GPL}$ = 2.5 μm, $t_{GPL}$ = 1.5 nm, $E_{GPL}$ = 1.01 TPa, $\rho_{GPL}$ = 1062.5 kg/m${}^{3}$, $v_{GPL}$ = 0.186, and $\alpha_{GPL}$ = −3.75 $\times 10^{-6}$$K^{-1}$ [22,44,45,46].

### 4.1. Validation and Convergence Study

**Figure 3:**
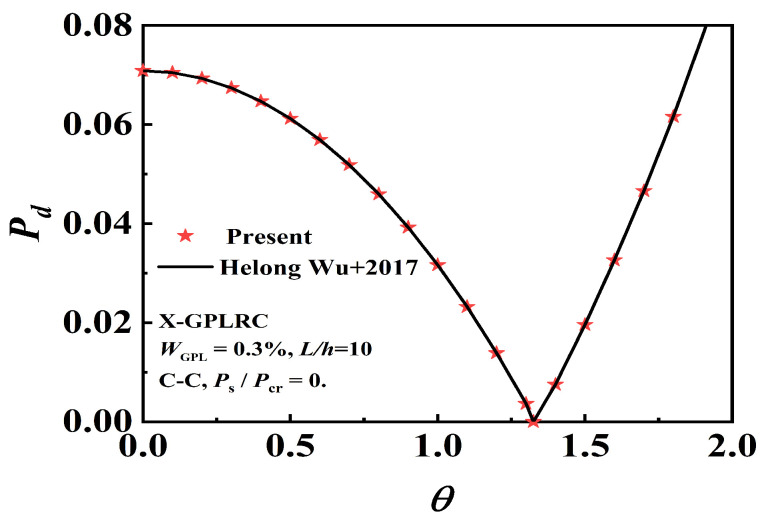
Comparison of the dynamic instability.

First, validation analysis is conducted. We adopt the degenerate forms of $K_{w}=0$, $K_{s}=0$, and ΔT=0 to compare and validate with references [25,47]. Table 1 and Table 2 compare the fundamental frequency and critical buckling load with the calculation results in reference [25]. Figure 3 verifies the dynamic instability of Wu et al. [47]. In general, our results are consistent with the existing results.

**Figure 4:**
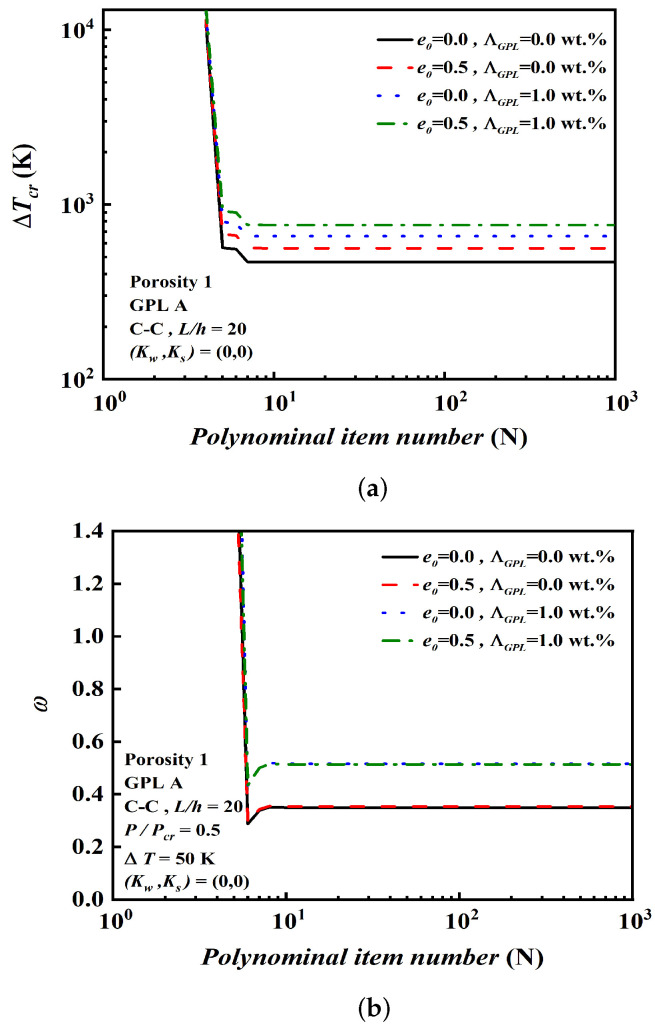
Determine polynomial item number (N). (**a**) Determine polynomial item number (N) of the critical buckling temperature rise. (**b**) Determine polynomial item number (N) of the dimensionless fundamental frequency.

Figure 4a,b separately show the convergence results of the critical buckling temperature rise and the dimensionless natural frequency under various conditions. When N=9, their values gradually approach a certain amount. Table 3 and Table 4 present the effect of the porosity coefficient $e_{0}$ and the total number of layers *n* on the critical buckling temperature rise and dimensionless natural frequency by DQM and the two-step perturbation method (TSPM). It turns out that the error between them is within 0.1%, and the accuracy and efficiency of the calculation results are verified again. Suppose *n* = 1000 is a continuous beam; it is found that a relative difference between *n* = 14 and *n* = 1000 is less than 1.5%. Considering the manufacturing process and manufacturing costs, *n* = 14 and N=9 are used in the following calculation. In addition, when *n* = 14 and N=9, the results show the natural frequency and critical buckling temperature rise are both increasing as $e_{0}$ increases.

### 4.2. Thermal Buckling

**Figure 5:**
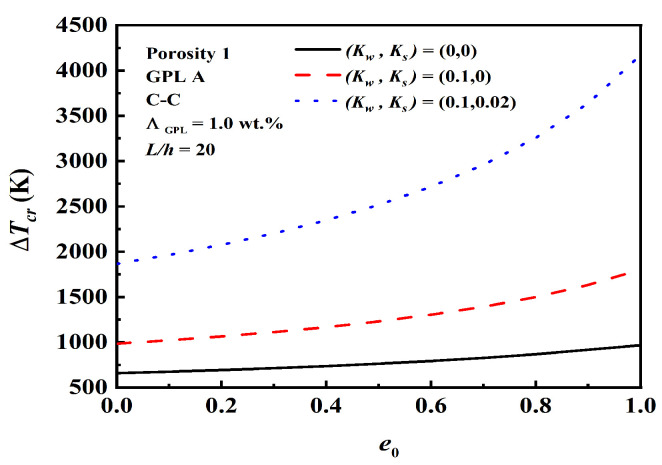
Effect of foundation stiffness for critical buckling temperature rise.

Figure 5 examines the effect of the foundation’s stiffness in critical buckling temperature rise. Where $\left(K_{w},K_{s}\right)=\left(0,0\right)$ stands for no foundation, $\left(K_{w},K_{s}\right)=\left(0.1,0\right)$ stands for Winkler foundation, and $\left(K_{w},K_{s}\right)=\left(0.1,0.02\right)$ stands for Pasternak foundation. As observed, the critical buckling temperature increases as the foundation stiffness increases. The shearing layer stiffness $K_{s}$ contributes to more enhancement than the Winkler foundation stiffness $K_{w}$.

**Figure 6:**
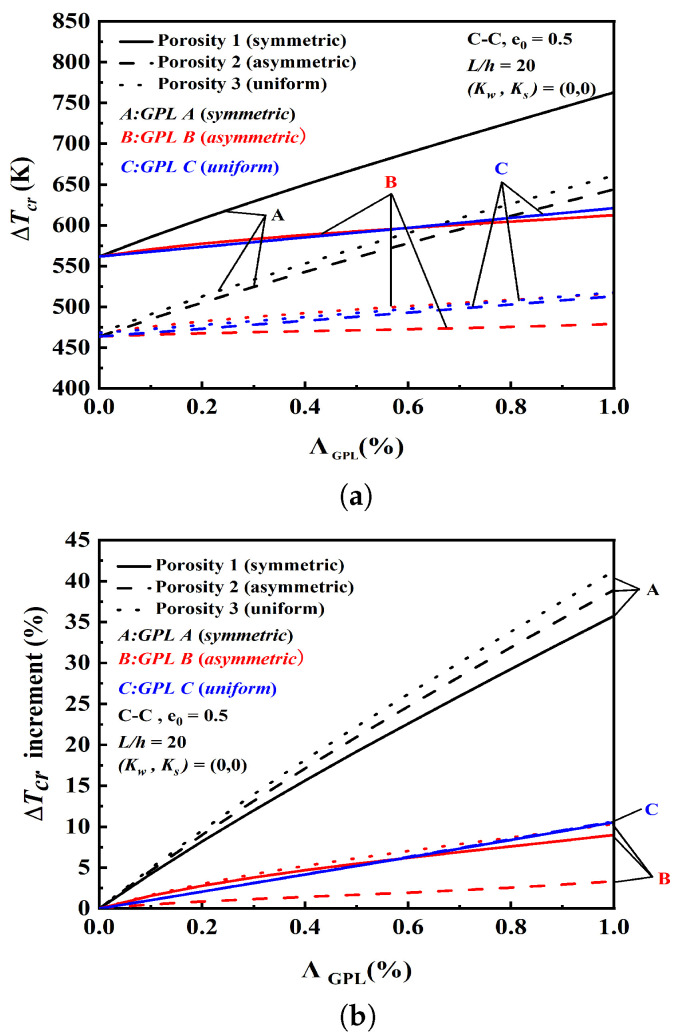
Effect of GPL weight fraction $\Lambda_{GPL}$ on critical buckling temperature rise and its percentage. (**a**) Effect of GPL weight fraction $\Lambda_{GPL}$ on critical buckling temperature rise. (**b**) Effect of GPL weight fraction $\Lambda_{GPL}$ on the percentage of critical buckling temperature rise.

Figure 6a,b show the critical buckling temperature rise and its percentage increment at GPL weight fraction $\Lambda_{GPL}$. The results identify that symmetric GPL A with porosity 1 provides the best reinforcement, which takes the largest critical buckling temperature rise of the nine models. In addition, GPL C plays no role in the critical buckling temperature rise for the three porosity distributions.

Table 5 illustrates the effect of boundary conditions and slenderness ratio L/h on critical buckling temperature rise. As expected, the C-C beam with a smaller slenderness ratio has a maximum critical buckling temperature rise. With the increment of L/h, the result shows a downward trend.

**Figure 7:**
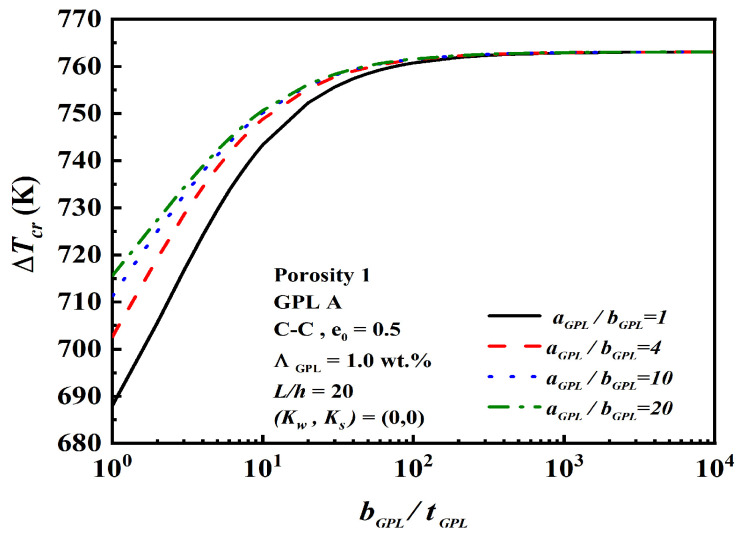
Effect of $a_{GPL}/b_{GPL}$ and $b_{GPL}/t_{GPL}$ of GPL nanofillers on the critical buckling temperature rise.

Figure 7 depicts the effect of geometry and size $a_{GPL}/b_{GPL}$ and $b_{GPL}/t_{GPL}$ on the critical buckling temperature rise. Larger $a_{GPL}/b_{GPL}$ and $b_{GPL}/t_{GPL}$ efficiently enhance the critical buckling temperature rise. Moreover, when $b_{GPL}/t_{GPL}$ reaches $10^{3}$, the critical buckling temperature rise reaches a certain level, it will no longer increase any more, and as $a_{GPL}/b_{GPL}$ increases, the change in critical buckling temperature rise becomes less and less obvious.

### 4.3. Thermo-Mechanical Vibration

**Figure 8:**
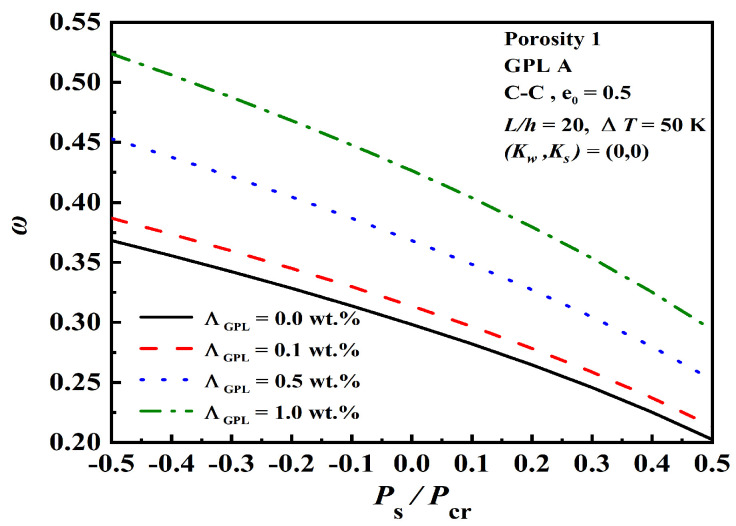
Effect of GPL weight fractions $\Lambda_{GPL}$ and normalized static axial force $P_{s}/P_{cr}$ on the dimensionless fundamental frequency.

Figure 8 presents the effect of GPL weight fractions $\Lambda_{GPL}$ and normalized static axial force $P_{s}/P_{cr}$ on the dimensionless fundamental frequency. The positive and negative values of $P_{s}/P_{cr}$ indicate the compressive force and tensile forces. $P_{cr}$ represents the critical buckling load at ΔT=0 K. As observed, increasing $\Lambda_{GPL}$ leads to better mechanical behavior.

Table 6 examines the effect of a normalized static axial force $P_{s}/P_{cr}$ on the dimensionless fundamental frequency under porosity distributions and GPL patterns. Due to compression forces compressing the beam, the free vibration frequency decreases when the compression force increases. Like thermal buckling, GPL A with porosity 1 has the highest free-vibration frequency.

**Figure 9:**
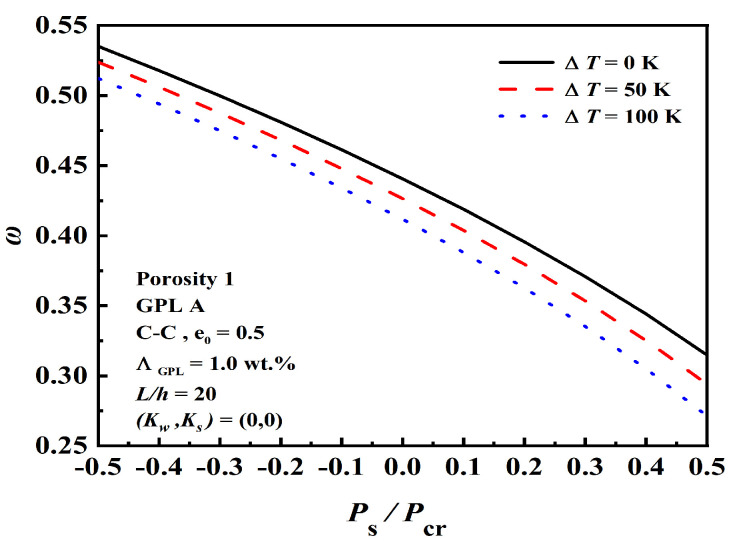
Effect of normalized static axial force $P_{s}/P_{cr}$ on the dimensionless fundamental frequency under initial thermal loading ΔT.

The effects of normalized static axial force $P_{s}/P_{cr}$ on the dimensionless fundamental frequency under initial thermal loading ΔT are shown in Figure 9. With the increment of initial thermal loading, the overall trend is downward. The changes in the dimensionless fundamental frequency were more apparent at larger values of $P_{s}/P_{cr}$.

**Figure 10:**
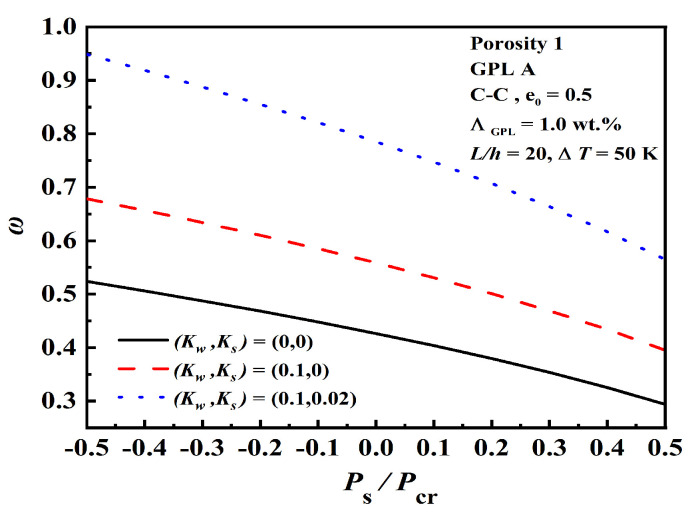
Effect of normalized static axial force $P_{s}/P_{cr}$ on the dimensionless fundamental frequency under various foundation stiffness.

Figure 10 reveals the effects of normalized static axial force $P_{s}/P_{cr}$ on the dimensionless fundamental frequency under various foundation stiffness. The dimensionless fundamental frequency increases as the foundation stiffness increases. Compared with compressive force, tensile force enhances the dimensionless fundamental frequency, which strengthens the stiffness of the beam.

### 4.4. Dynamic Instability

**Figure 11:**
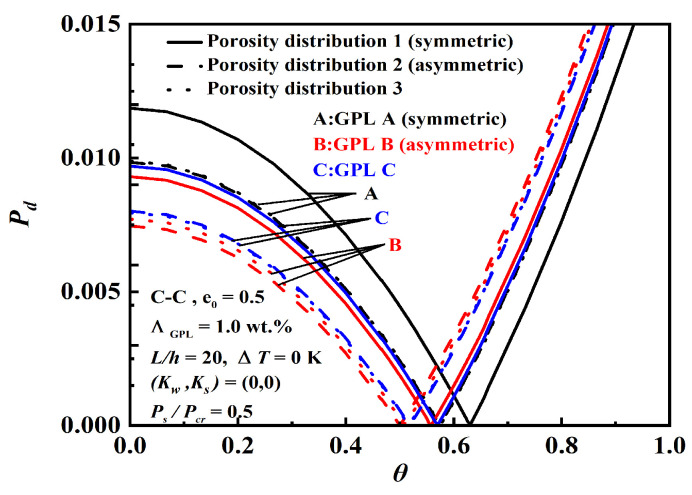
Effect of porosity distributions and GPL patterns on dynamic instability.

Figure 11 illustrates the effect of porosity distributions and GPL patterns on dynamic instability. Like thermal buckling and thermo-mechanical vibration, GPL A with porosity 1 has the largest origin and the narrowest unstable region among the nine models. Then, it is the best-enhanced model because the beam has a smaller pore distribution and more GPL in the top and bottom layers, which is where the normal bending stress is highest.

**Figure 12:**
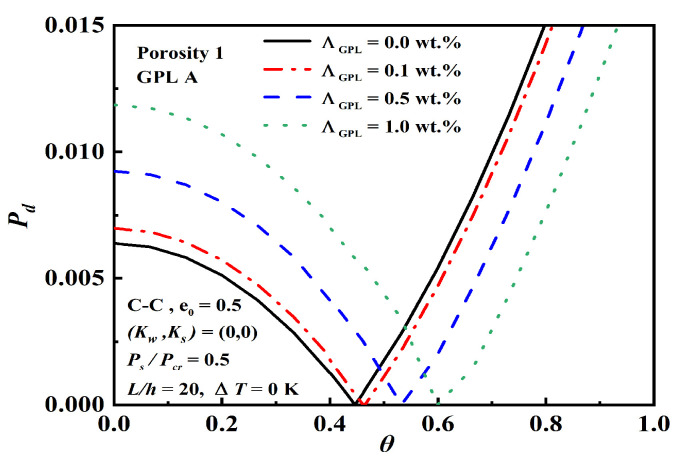
Effect of GPL weight fractions $\Lambda_{GPL}$ on dynamic instability.

Figure 12 depicts the effect of GPL weight fractions $\Lambda_{GPL}$ on dynamic instability. It is found that the origin becomes larger and the unstable region becomes narrower with the increment of $\Lambda_{GPL}$, which indicates that the addition of GPL nanofillers effectively raises the stiffness of the beam.

**Figure 13:**
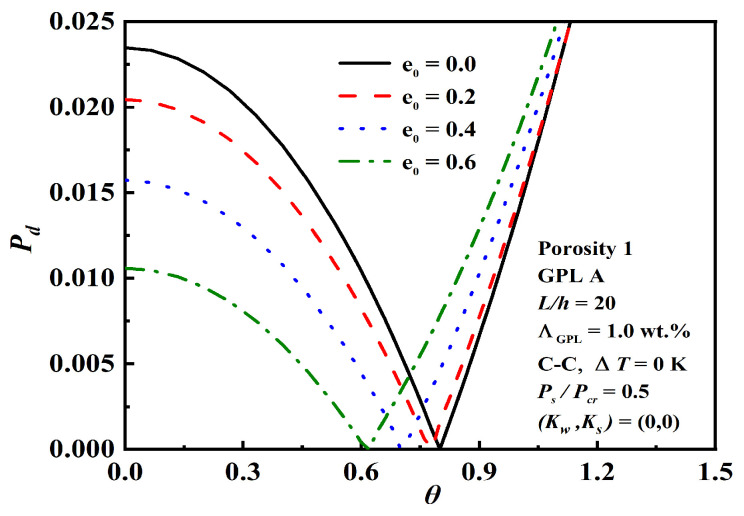
Effect of the porosity coefficient $e_{0}$ on dynamic instability.

Figure 13 investigates the effect of the porosity coefficient $e_{0}$ on dynamic instability. As observed, the increment in $e_{0}$, which means that the beam has larger pores and a denser distribution of pores, causes a reduction in beam stiffness, the origin becomes lower, and the unstable region becomes wider.

**Figure 14:**
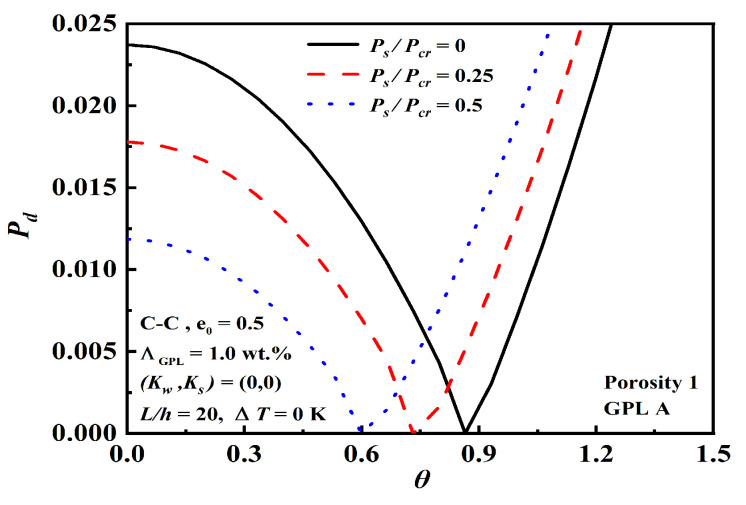
Effect of static axial compressive force $P_{s}/P_{cr}$ on dynamic instability.

**Figure 15:**
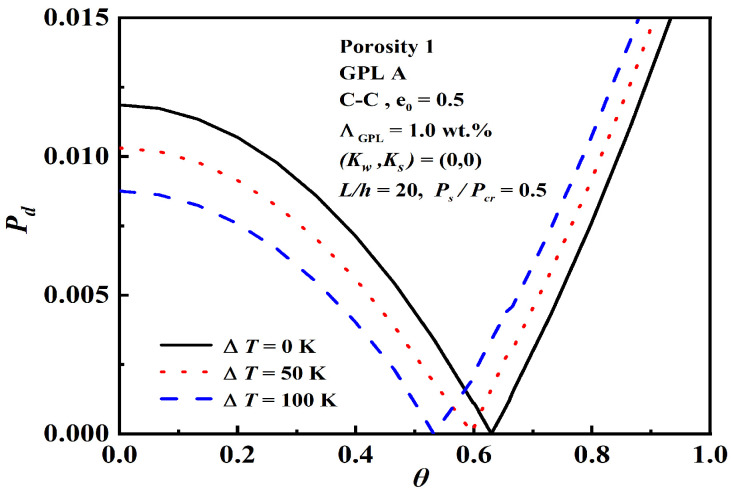
Effect of initial thermal loading ΔT on dynamic instability.

The effect of static axial compressive force $P_{s}/P_{cr}$ and initial thermal loading ΔT on dynamic instability are shown in Figure 14 and Figure 15. Due to the change in $P_{s}/P_{cr}$ and ΔT, the beam produces compression force, thus reducing the beam stiffness. As $P_{s}/P_{cr}$ and ΔT decrease, the origin increases and the unstable region narrows. $P_{s}/P_{cr}$ is able to achieve better structural stiffness, even more significantly than ΔT at the dynamic instability.

**Figure 16:**
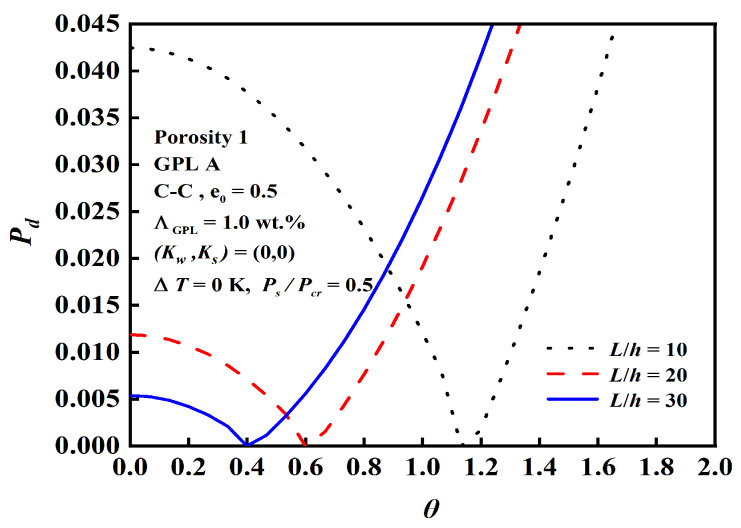
Effect of the slenderness ratio L/h on dynamic instability.

**Figure 17:**
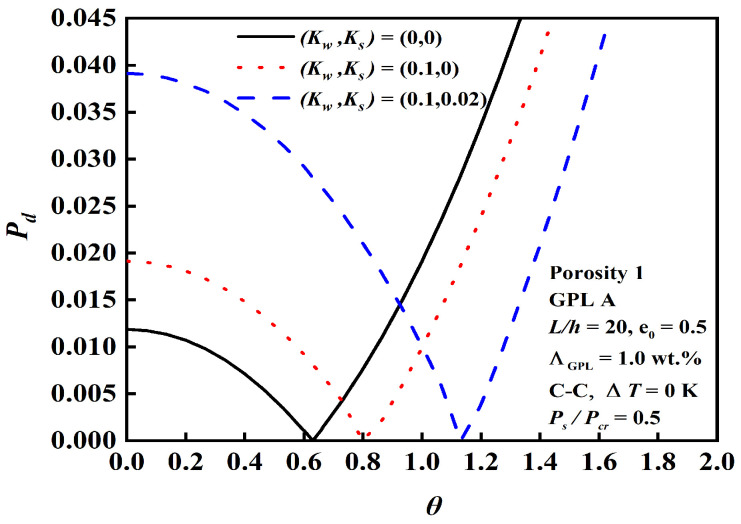
Effect of the foundation stiffness on dynamic instability.

**Figure 18:**
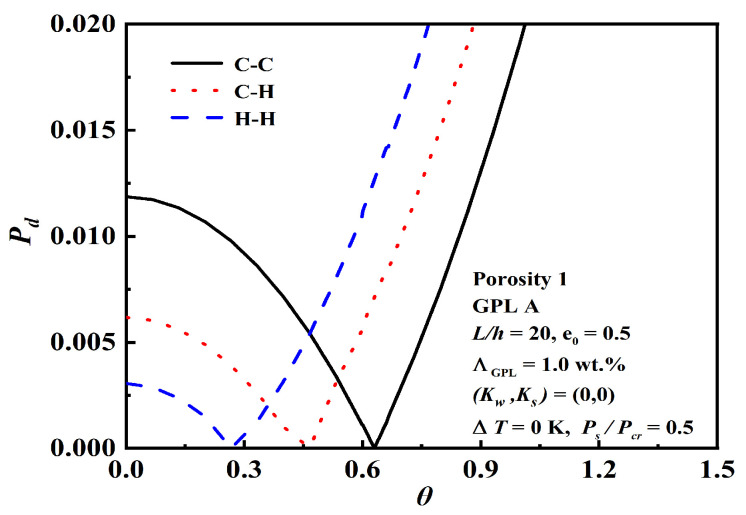
Effect of the boundary conditions on dynamic instability.

The effect of the slenderness ratio L/h, foundation stiffness, and boundary conditions on dynamic instability is demonstrated in Figure 16, Figure 17 and Figure 18. The results depict that the C-C beam with a smaller slenderness ratio on a Pasternak elastic foundation has a bigger origin and narrower unstable region, and the slenderness ratio and foundation stiffness have more obvious effects than the boundary conditions.

**Figure 19:**
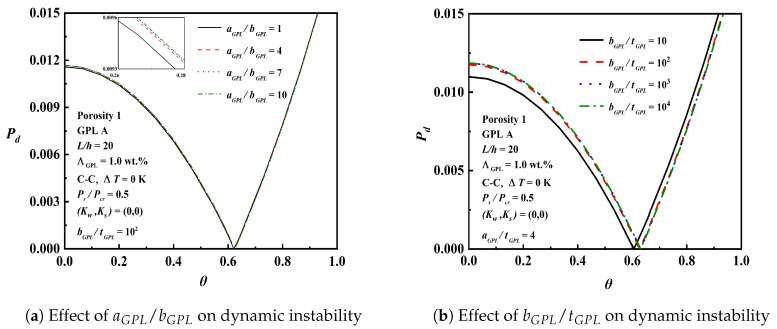
Effect of $a_{GPL}/b_{GPL}$ and $b_{GPL}/t_{GPL}$ on dynamic instability.

Figure 19 examines the effect of the geometry and size $a_{GPL}/b_{GPL}$ and $b_{GPL}/t_{GPL}$ on dynamic instability. For a given $b_{GPL}/t_{GPL}=10^{2}$, as $a_{GPL}/b_{GPL}$ increases, the change in the unstable region is insignificant, and the effect is tiny. For a given $a_{GPL}/b_{GPL}=4$, the change in $b_{GPL}/t_{GPL}$ from 10 to $10^{2}$ has an obvious effect on the dynamic instability, but the effect of $b_{GPL}/t_{GPL}$ over $10^{2}$ is negligible. The results show that when the GPL contains less than a single graphene layer, it is better to reinforce the stiffness of the beam and enhance the mechanical behavior.

## 5. Conclusions

The effect of GPL nanofillers on FGP composite beams under thermal environments, thermal buckling, thermo-mechanical vibration, and dynamic instability are investigated. Among them, weight fraction, normalized static axial force, porosity coefficient, dispersion pattern, boundary conditions, initial thermal loading, geometry and size, foundation stiffness, and slenderness ratio are studied. The following conclusions are obtained:

• Porosity 1 reinforced by GPL A of the beam has the biggest value of critical buckling, temperature rise, dimensionless fundamental frequency, and the origin of dynamic instability. The non-uniform, symmetric porosity distribution and GPL pattern have the strongest enhancement.
• The porosity coefficient has an important influence on thermal buckling, thermo-mechanical vibration, and dynamic instability. When the porosity coefficient grows, the origin of the dynamic instability shows a decreasing trend, but the dimensionless fundamental frequency and critical buckling temperature rise both increase.
• The addition of GPL nanofillers can enhance the beam stiffness significantly, and the mechanical performance is enhanced with $\Lambda_{GPL}$ increases.
• The values of thermo-mechanical vibration and dynamic instability decrease with normalized static axial force and initial thermal loading increase.
• Winkler and Pasternak foundations both strengthen the stiffness of the beam. It is noted that shearing layer stiffness has a better enhancement effect than Winkler foundation stiffness.

## Figures and Tables

**Table 1:** The dimensionless fundamental frequency of C-C beams (porosity 1, GPL A, L/h=20).

$\Lambda_{\mathrm{GPL}}$	*n*	$e_{0}=0$		$e_{0}=0.2$		$e_{0}=0.4$		$e_{0}=0.6$	
		**Ref. [25]**	**Present**	**Ref. [25]**	**Present**	**Ref. [25]**	**Present**	**Ref. [25]**	**Present**
1 wt.%	2	0.3376	0.3376	0.3217	0.3217	0.3042	0.3042	0.2845	0.2845
	6	0.4390	0.4390	0.4336	0.4336	0.4289	0.4289	0.4259	0.4259
	10	0.4464	0.4464	0.4421	0.4421	0.4388	0.4388	0.4372	0.4372
	14	0.4484	0.4484	0.4444	0.4444	0.4415	0.4415	0.4403	0.4403
	18	0.4492	0.4492	0.4454	0.4454	0.4426	0.4426	0.4416	0.4416
	10,000	0.4505	0.4505	0.4468	0.4468	0.4442	0.4442	0.4436	0.4436
0 wt.%	14	0.3159	0.3159	0.3134	0.3134	0.3121	0.3121	0.3128	0.3128
	10,000	0.3167	0.3167	0.3144	0.3144	0.3132	0.3132	0.3142	0.3142

**Table 2:** The dimensionless critical buckling load of C-C beams (porosity 1, GPL A, L/h=20).

$\Lambda_{\mathrm{GPL}}$	*n*	$e_{0}=0$		$e_{0}=0.2$		$e_{0}=0.4$		$e_{0}=0.6$	
		**Ref. [25]**	**Present**	**Ref. [25]**	**Present**	**Ref. [25]**	**Present**	**Ref. [25]**	**Present**
1 wt.%	2	0.008550	0.008550	0.007218	0.007219	0.005917	0.005918	0.004647	0.004647
	6	0.014323	0.014324	0.013057	0.013058	0.011784	0.011784	0.010486	0.010486
	10	0.014798	0.014798	0.013572	0.013573	0.012333	0.012334	0.011063	0.011063
	14	0.014929	0.014929	0.013714	0.013715	0.012486	0.012486	0.011224	0.011224
	18	0.014982	0.014983	0.013773	0.013774	0.012549	0.012549	0.011290	0.011290
	10,000	0.015065	0.015066	0.013863	0.013864	0.012645	0.012646	0.011392	0.011392
0 wt.%	14	0.007946	0.007947	0.007316	0.007316	0.006693	0.006693	0.006076	0.006076
	10,000	0.007986	0.007986	0.007362	0.007362	0.006745	0.006746	0.006135	0.006135

**Table 3:** The critical buckling temperature rise of H-H beams (porosity 1, GPL A, L/h=20, $K_{w}=K_{s}=0$).

$\Lambda_{\mathrm{GPL}}$	*n*	$e_{0}=0$			$e_{0}=0.2$			$e_{0}=0.4$			$e_{0}=0.6$		
		**DQM**	**TSPM**	**Error**	**DQM**	**TSPM**	**Error**	**DQM**	**TSPM**	**Error**	**DQM**	**TSPM**	**Error**
1 wt.%	2	97.7638	97.7646	0.0008%	97.5424	97.5433	0.0009%	97.2608	97.2616	0.0008%	96.8839	96.8847	0.0008%
	6	162.4627	162.4641	0.0009%	170.1854	170.1868	0.0008%	179.9406	179.9421	0.0008%	192.7597	192.7613	0.0008%
	10	167.7434	167.7448	0.0008%	176.4998	176.5012	0.0008%	187.5116	187.5131	0.0008%	201.8757	201.8774	0.0008%
	14	169.1999	169.2013	0.0008%	178.2459	178.2474	0.0008%	189.6084	189.6100	0.0008%	204.4014	204.4031	0.0008%
	18	169.7995	169.8009	0.0008%	178.9652	178.9667	0.0008%	190.4726	190.4742	0.0008%	205.4423	205.4440	0.0008%
	1000	170.7175	170.7189	0.0008%	180.0671	180.0686	0.0008%	191.7968	191.7984	0.0008%	207.0375	207.0392	0.0008%
0 wt.%	14	119.4444	119.4454	0.0008%	127.2565	127.2576	0.0009%	137.5587	137.5598	0.0008%	151.8699	151.8712	0.0008%
	1000	120.0523	120.0533	0.0008%	128.0345	128.0356	0.0009%	138.5566	138.5578	0.0009%	153.1614	153.1627	0.0008%

**Table 4:** The dimensionless fundamental frequency of H-H beams (porosity 1, GPL A, L/h=20, $K_{w}=K_{s}=0$, ΔT=50K).

$\Lambda_{\mathrm{GPL}}$	*n*	$e_{0}=0$			$e_{0}=0.2$			$e_{0}=0.4$			$e_{0}=0.6$		
		**DQM**	**TSPM**	**Error**	**DQM**	**TSPM**	**Error**	**DQM**	**TSPM**	**Error**	**DQM**	**TSPM**	**Error**
1 wt.%	2	0.1053	0.1051	0.1899%	0.1002	0.1000	0.1996%	0.0946	0.0944	0.2114%	0.0882	0.0881	0.1134%
	6	0.1639	0.1636	0.1830%	0.1636	0.1633	0.1834%	0.1638	0.1635	0.1832%	0.1649	0.1645	0.2426%
	10	0.1679	0.1676	0.1787%	0.1682	0.1678	0.2378%	0.1690	0.1686	0.2367%	0.1707	0.1704	0.1757%
	14	0.1690	0.1687	0.1775%	0.1694	0.1691	0.1771%	0.1704	0.1700	0.2347%	0.1723	0.1720	0.1741%
	18	0.1695	0.1691	0.2360%	0.1699	0.1696	0.1766%	0.1710	0.1706	0.2339%	0.1730	0.1726	0.2312%
	1000	0.1702	0.1698	0.2350%	0.1707	0.1703	0.2343%	0.1718	0.1715	0.1746%	0.1740	0.1736	0.2299%
0 wt.%	14	0.1078	0.1076	0.1855%	0.1094	0.1092	0.1828%	0.1117	0.1114	0.2686%	0.1151	0.1148	0.2606%
	1000	0.1083	0.1081	0.1847%	0.1100	0.1097	0.2727%	0.1123	0.1121	0.1781%	0.1158	0.1156	0.1727%

**Table 5:** Effect of boundary conditions and slenderness ratio L/h on critical buckling temperature rise. (Porosity 1, GPL A, $e_{0}=0.5$, $\Lambda_{GPL}=1.0\;\mathrm{wt.\%}$, $K_{w}=K_{s}=0$).

BC	L/h=10	L/h=15	L/h=20	L/h=25	L/h=30
C-C	2730.8457	1316.1778	762.8928	495.2309	346.6018
C-H	1487.9949	693.6980	397.0135	256.1591	178.6797
H-H	762.8528	346.5832	196.4819	126.2065	87.8172

**Table 6:** Effect of normalized static axial force $P_{s}/P_{cr}$ on the dimensionless fundamental frequency under porosity distributions and GPL patterns ($e_{0}=0.5$, $\Lambda_{GPL}$ = 1.0 wt.%, C-C, L/h=20, ΔT=0K, $K_{w}=K_{s}=0$).

Multilayer Beam	1A	2A	3A	1B	2B	3B	1C	2C	3C
$P_{s}/P_{cr}=0.00$	0.4264	0.3863	0.3863	0.3748	0.3318	0.3389	0.3829	0.3452	0.3441
$P_{s}/P_{cr}=0.25$	0.3668	0.3314	0.3314	0.3213	0.2830	0.2896	0.3283	0.2949	0.2941
$P_{s}/P_{cr}=0.50$	0.2939	0.2641	0.2641	0.2556	0.2227	0.2287	0.2613	0.2329	0.2323

## Data Availability

The data presented are available in this article.
